# Recent Progress in Magnetron Sputtering Technology Used on Fabrics

**DOI:** 10.3390/ma11101953

**Published:** 2018-10-12

**Authors:** Xue-Qiang Tan, Jian-Yong Liu, Jia-Rong Niu, Jia-Yin Liu, Jun-Ying Tian

**Affiliations:** 1 College of Textiles, Tianjin Polytechnic University, Tianjin 300387, China; tan_xueqiang@126.com (X.-Q.T.); liujiayin6@126.com (J.-Y.L.); tjy6818@163.com (J.-Y.T.); 2 Key Laboratory of Advanced Textile Composites Ministry of Education, Tianjin Polytechnic University, Tianjin 300387, China

**Keywords:** magnetron sputtering, target materials, fabrics, nano-film, functions and characterization

## Abstract

The applications of magnetron sputtering technology on the surface coating of fabrics have attracted more and more attention from researchers. Over the past 15 years, researches on magnetron sputtering coated fabrics have been mainly focused on electromagnetic shielding, bacterial resistance, hydrophilic and hydrophobic properties and structural color etc. In this review, recent progress of the technology is discussed in detail, and the common target materials, technologies and functions and characterization of coated fabrics are summarized and analyzed. Finally, the existing problems and future prospects of this developing field are briefly proposed and discussed.

## 1. Introduction

Nano-films are widely used in electronics, textiles, biomedicine, ceramics and other fields [1,2,3,4,5,6]. Fabrics coated with Nano film are usually prepared through chemical vapor deposition [7], chemical deposition [8,9], sol-gel [10] method and magnetron sputtering [11]. Among them, the magnetron sputtering method has the advantages of controllable film thickness, high purity, high speed and low temperature, favorable adhesion, easy operation, and environmental friendliness, etc. [12,13].

Magnetron sputtering technology can deposite metal or non-metal films on the substrate surface of textiles such as polyester, cotton, linen, silk, wool, polyamide, polylactic acid and polypropylene through selecting the appropriate sputtering process, different target materials and ambient gases [14,15]. The main structures of the textiles substrate include woven fabrics, knitted fabrics and non-woven fabrics. The sputtering target materials are metals such as Cu, Ti, Ag, Al, W, Ni, Sn, Pt or non-metals such as Si, graphite as well as metal oxides such as TiO_2_, Fe_2_O_3_, WO_3_, ZnO and non-metal oxide such as SiO_2_. It can also deposit ceramic materials, and single or multi-layer composite nano-films formed with polymers such as polyimide, and polytetrafluoroethylene etc. It can not only endow fabrics with single or compound functions such as electromagnetic shielding, UV protection, anti-static, antibacterial, conductive or waterproof properties, etc., but also obtain structural colors through interference and diffraction characters of the nano-films [16,17,18,19,20,21,22,23,24,25,26,27,28]. Therefore, the magnetron sputtering coating nano-films technology used on fabrics has been vigorously developed and applied [22,29].

The summary of magnetron sputtering target materials commonly used and coated fabrics’ functionalities are shown in Table 1.

**Figure 1:**
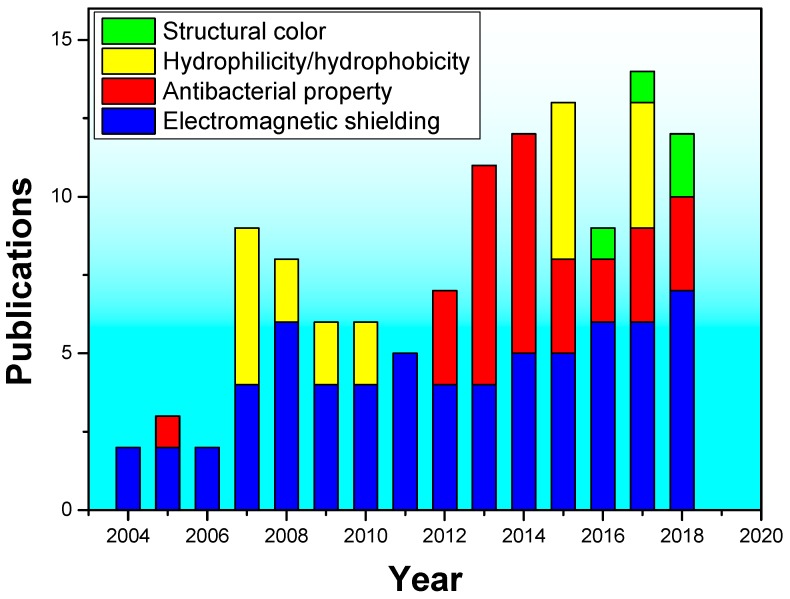
The amount of literatures on magnetron sputtering functional textiles in recent years.

**Figure 2:**
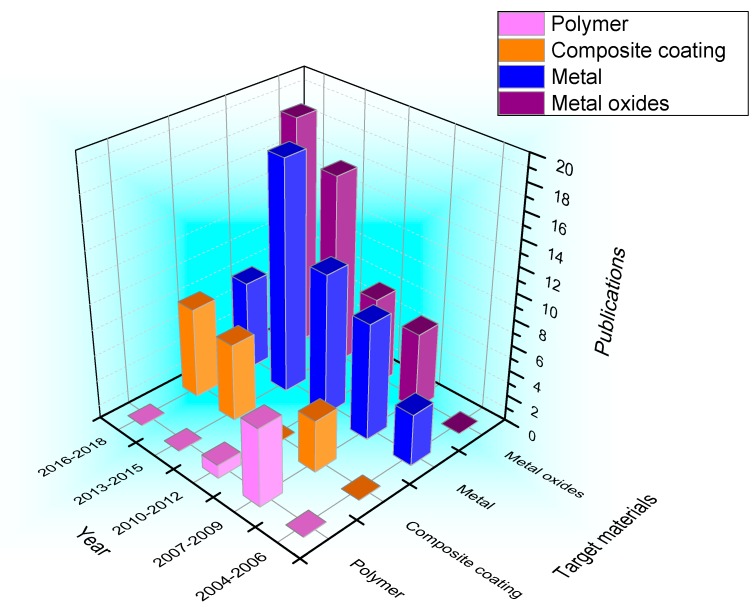
The amount of literatures on magnetron sputtering coated fabrics target materials in recent years.

The number of references related to the functionality and target materials of magnetron sputtering coated fabrics in the last 15 years are illustrated in Figure 1 and Figure 2, respectively (the year of 2018 is from January to July). In Figure 1, it can be found that the number of literatures on magnetron sputtering coated fabrics from 2004 to 2018 increased year by year. Among them, the research on preparation of electromagnetic shielding fabrics is most extensive, and a certain number of research papers are published every year. However, papers on hydrophilic and hydrophobic-coated fabrics have been published on and off since 2007. The application to prepare antibacterial fabrics has been a continuing research content since 2012. The research papers of structural color of coated fabrics have only been published in the last three years because of the characteristics of saving water resources and environmental protection. It is also an important trend in the field of textile printing and dyeing research in the future. In Figure 2, it can be found that the commonly used target materials on magnetron sputtering coated fabrics were metals, metal oxides, polymer materials and composite coatings from 2004 to 2018. The research on polymer materials as target materials has been mainly focused on 2007–2009, the polymer target materials are commonly used for hydrophobic finishing and the amount of literatures is small. The application of metal target materials to obtain electrical conductivity, shielding efficiency had gradually increased in 2004–2015, but decreased from 2016. However, the metal oxides as target materials have been researched increasingly. In addition, it is necessary to pay attention to the increasing trend of composite sputtering using various target materials. Although the number of studies is not much, this method can combine the advantages of various target materials to impart fabrics multiple functions at the same time. So, it is a possible research focus for the future.

## 2. Application of Fabrics Coated with Magnetron Sputtering Nano Film

### 2.1. Nano Metal Film

Through magnetron sputtering technology, metallization of the fabric surface can be realized [30]. Surface metallization endows fabrics and fibers multiple functions [31], such as antibacterial, antistatic, anti-ultraviolet, electrical conductivity and electromagnetic shielding properties, etc. The metalized fabrics can be used for medical materials, anti-eavesdrop materials, decorative materials, radar reflective materials, military tents and pregnancy protection cloth, etc. [32,33,34].

#### 2.1.1. Nano Cu Film

Nano Cu film-coated fabrics have excellent electromagnetic shielding, electrical conductivity, UV resistance and antibacterial properties [35,36].

The nano Cu film fabrics coated by magnetron sputtering had favorable antibacterial property against *Escherichia coli*. Under the same sputtering conditions, bacteriostatic rates against *Escherichia coli* of the nano Cu film deposited by high-power pulse sputtering was more than three times higher than that deposited through direct current magnetron sputtering [37]. Scholz et al. [38] sputtered Cu and Ag on fabrics, respectively. Compared with Cu and Ag nano-films, the antibacterial properties of Cu exceeded that of Ag. Although the Cu coated fabric has favorable antibacterial effect, it currently only act on several limited kinds of bacteria, such as *Staphylococcus aureus* and *Escherichia coli*. It is difficult to achieve broad spectrum antibacterial effects [39]. 

**Figure 3:**
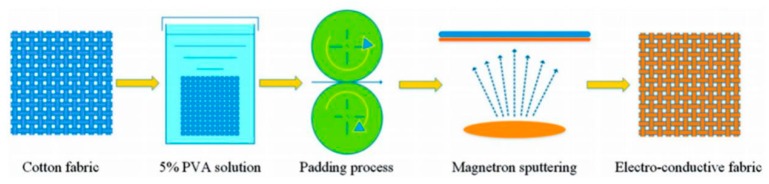
Schematic of the preparation of PVA impregnated cotton fabric by padding and sputtering. (Reprinted from Reference [43] with permission).

Magnetron sputtering nano Cu film coated fabrics have so favorable electromagnetic shielding performance. Kim et al. [40] used direct current magnetron sputtering method to deposite Ag nano-film firstly on polyimide substrate, and then to deposite a layer of Cu nano-film. The electromagnetic shielding efficiency of the prepared composite fabric was greater than 55 dB at a frequency range of 10 MHz to 1.3 GHz. At ambient temperature, Wang L. et al. [41] deposited nano Cu film on the surface of polyester fabric. The coated fabric not only had favorable shielding effectiveness and electrical conductivity, but also excellent shielding effect on ultraviolet light. Meanwhile the shielding effect is also affected by the substrates, especially the porosity of the substrate. Huang et al. [41] used radio frequency (RF) magnetron sputtering method to sputter nano Cu film on substrates with different structures. Among them, the coated fabric with the lowest porosity based on polyester non-woven fabric had the best electromagnetic shielding effect, and the shielding efficiency was up to 40 dB at a frequency range of 30 MHz to 1.5 GHz. In order to reduce the porosity of the fabric [42], the cotton fabric with large porosity was first coated with polyvinyl alcohol (PVA), then the Cu or Ti were sputtering deposited on the treated fabric substrates respectively. The schematic of the preparation of PVA impregnated cotton fabric by padding and sputtering method is shown in Figure 3. The results showed that electrical conductivity and electromagnetic shielding properties of them were improved, and electromagnetic shielding efficiency of the Cu coated samples reaches 30 dB at a frequency range of 300 kHz to 1.8 GHz.

When the Cu film coated on fabric reaches a certain thickness, it also perform better in aspects of infrared and ultraviolet shielding properties. Cu film with thickness of 200 nm was deposited onto cotton fabric by magnetron sputtering technology [43]. The physical properties of the coated fabrics that are evaluated include the infrared emissivity, reflection rate and ultraviolet protection factor (UPF) value. It was found that the Cu coated fabric samples had an infrared reflection rate of 20–30%, infrared emissivity of about 0.7 and UPF value of 273. The Cu-coated fabric samples therefore provide excellent UV radiation protection and good infrared shielding, which make them promising materials for sunlight management textiles.

In summary, the Cu film coated fabrics can achieve favorable electromagnetic shielding, UV resistance and anti-infrared properties. However, it is difficult to achieve shielding effectiveness of greater than 60 dB for general electromagnetic shielding fabrics. There are few researches on microwave electromagnetic shielding at a higher frequency range of 10–18 GHz. In addition, we take into account the phenomenon of photonic band gap generated by the special periodic structure of photonic crystals, it is worthwhile to study whether the periodic multilayer metal films can selectively shield electromagnetic waves with different frequencies.

#### 2.1.2. Nano Ag Film

In metals, Ag has the best thermal conductivity and electrical conductivity and it has soft texture, ductility, good wear resistance and antibacterial properties [44,45,46]. Montazer et al. [47] sputtered a variety of metal films on nylon 6 fabrics, and it was found that the electric conductivity of the samples from strong to weak was Ag film, Cu film, and Al film, which was consistent with the order of electric conductivity of metal Ag, Cu and Al.

The square resistance of nano Ag films deposited by magnetron sputtering can reach 10^−2^ Ω orders, shielding efficiency is as high as 60–80 dB. Therefore, Ag film coated fabric can endow excellent electromagnetic shielding and electrical conductivity properties to textiles [48]. Even if very thin Ag films of 2–20 nm are sputtered on the PET fabric surface, the coated fabrics can achieve favorable electromagnetic shielding effect and excellent electrical conductivity property [49].

The experiments by Montazer et al. [47] also proved that the electrical conductivity of the coated fabric was related to the film’s density. Compared with the traditional metal printing, the nano Ag particles coated by magnetron sputtering dispersed more evenly on the surface of fabric, better reflecting electromagnetic waves, and the fabric damage is very little [23]. Experiments by Du et al. [50] also proved that the better the continuity and compaction of the nano Ag film was, the higher the electromagnetic shielding efficiency was.

Metal Ag is also widely used to prepare antibacterial fabrics. Before sputtering deposition, low temperature plasma pretreatment can effectively improve the adhesion of the films. The washing fastness was up to five grades, and the samples had favorable antibacterial activity against *Staphylococcus aureus* and *Escherichia coli*. After 20 times washing, the original antibacterial effect was still maintained and the mechanical properties of the original fabric were also maintained [18]. Rtimi [51] sputtered ZrNO and Ag on PET fabrics to prepare antibacterial fabrics with ZrNO-Ag composite films, compared with the single layer film, the antibacterial effect of the composite film on Escherichia coli was significantly enhanced.

In addition, it is also possible to use the properties of high scattering and reflectivity of Ag to shield ultraviolet rays. When the sputtering pressure was 0.3 Pa, polyester fabric sputtered with Ag had the best UV resistance property [52]. Depositing nano Ag film on cotton fabric can also obtain favorable infrared resistance and hydrophobic properties. In some research the infrared reflectivity was up to 18% [53,54].

#### 2.1.3. Nano Ti Film

Metal Ti has the characteristics of light weight, high strength and biological compatibility etc. [55,56,57]. For example, Esen et al. deposited metal Ti films on a polyamide/cotton fabric (The textile material is composed of cotton and polyamide in different ratios) to develop a kind of electromagnetic wave absorbing fabric, which can be applied in radio communication and radar aspects [58]. Moreover, it is well known that the metal Ti would not cause any allergic reaction to human skin.

#### 2.1.4. Nano Al film

Metal Al has favorable physical and chemical properties such as corrosion resistance, thermal conductivity and electrical conductivity, etc. [59,60,61]. Sputtering Al films on the surface of fabrics can yield an impart effect, such as electrical conductivity, electromagnetic shielding and UV resistance to them.

Zhimin et al. [62] deposited nano Al films with different thickness on PET substrates. With the increase of film thickness, the electrical conductivity and electromagnetic shielding performance of coated fabrics were significantly improved. In addition, the theoretical calculation and transfer matrix method were used to verify that electrical conductivity had a significant effect on the microwave absorption performance. Bandorf et al. [63] sputtered Al on polyester fabric to prepare a uniform and compact film. With the increase of sputtering peak current, the adhesion of metal particles on the fabric surface was significantly improved, and the coated fabric had favorable UV shielding property.

In addition, it can also impart a certain water-repellent property for textiles to deposite Al film on fabric surface. Shahidi et al. [64] sputtered nano Al film on cotton fabrics. Water-repellent property of the fabrics was increased with the extension of sputtering time. Water-repellent property reached an optimum value when the fabric was sputtered for 30 min.

#### 2.1.5. Other Nano Metal Films

There was not much research on other metals used in textile sputtering processing except for Ni and Pt. Yuen et al. [65] sputtered metal Ni film on the surface of polyester fabrics. The coated fabric was significantly improved with hydrophilicity and ultraviolet shielding function. Shahidi et al. [66] sputtered metal Pt film on the polyester fabrics’ surface. The dyeing fastness of madder and henna on the polyester fabrics were improved by 4–5 grades. The mechanism was that metal ions as the central ions could coordinate with the fiber and the dyes.

### 2.2. Nano Metal Oxide Films

The deposition of metal oxide on the surface of the fabrics can endow them with antibacterial property, anti-static, gas sensitivity, ultraviolet resistance, electromagnetic shielding property, electrical conductivity and other properties [67,68]. For example, the semiconductor ceramic film materials such as SnO_2_, ZnO, Al_2_O_3_ and other oxides [69,70,71,72]. They have gas-sensitivity, therefore they could selectively adsorb gases. When their surface free energy was changed, the electrical conductivity was varied accordingly. So, it could be used to judge the kind of gases and measure their concentration. For example, oxygen, methane, ethylene and other gases.

#### 2.2.1. Nano TiO_2_ Film

Sputtering deposition of nano TiO_2_ film on fabrics mainly imparts favorable UV resistance and photocatalytic function to them, as well as additional functions such as antibacterial property, formaldehyde adsorption and hydrophilicity [73,74,75].

When TiO_2_ [76] was used as an ultraviolet shielding agent, the performance of the rutile type was more effective than that of the anatase type. As the nano TiO_2_ particles size increased, the nano film became more uniform and compact. When the film thickness increased to a critical value, the UV-resistant and photocatalytic properties of the coated fabrics could achieve the best effect. Zgura et al. [75] sputtered nano TiO_2_ film on polylactic acid (PLA) fabric. As the sputtering pressure increased, the photocatalytic property was improved, and degrading effect of methylene blue was more effective. Zhang [77], Rtimi et al. [78] sputtering deposited nano TiO_2_ film on the surface of non-woven fabric. Due to its unique photocatalytic property, it could obtain anti-fouling and deodorizing functions.

In order to improve the photocatalytic activity and enhance the antibacterial effect, Rtimi et al. sputtered nano TiON and TiON/Ag [79] films on polyester fabrics. When the thickness of the TiON film was 70 nm, *Escherichia coli* was completely deactivated in 120 min. If the Ag film was sputtered furthermore, *Escherichia coli* was deactivated quickly within 55 min on the TiON/Ag composite. Rtimi et al. also sputtered nano TiN and TiN/Ag [80] films on polyester fabrics. It was found that the rate of bacteria deactivation became faster for Ag enhanced the photocatalytic activity of TiN in TiN/Ag film.

**Figure 4:**
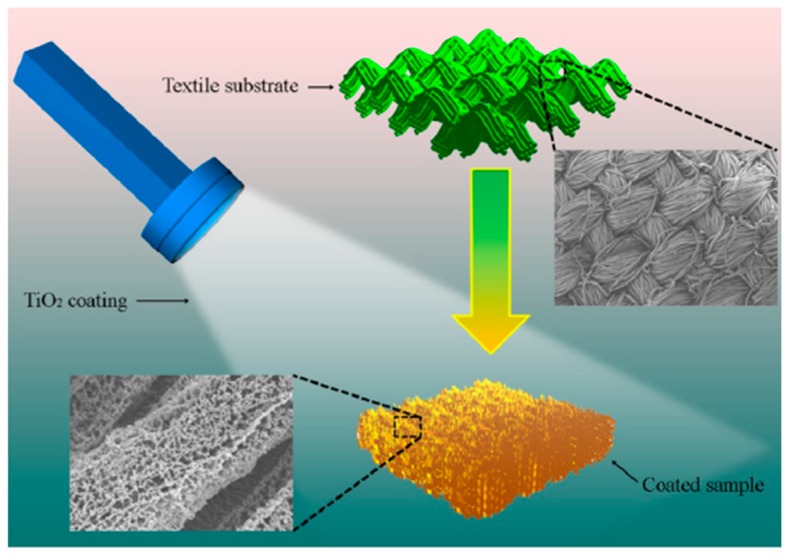
Scheme of the coating process. (Reprinted from Reference [84] with permission).

When polyacrylonitrile (PAN) and polyurethane (PU) composite materials (mass ratio of PAN/PU was 8/2) substrate was deposited with Nano TiO_2_ film [81], it not only had excellent UV resistance (UPF value was 148.5), but also good super-hydrophobility (contact angle was 152.1°). Its super-hydrophobic property was mainly due to rough surface structure was constructed on the fibers’ surface. For example, nano TiO_2_ films were deposited on cotton and polyester fabrics and the TiO_2_ films on fabric’s surface had unique high porosity and amorphous structure [82]. Its coating scheme was shown in Figure 4. With the increasement of sputtering time, the surface tension of the coated fabric was improved. Its waterproof property was improved significantly [83].

#### 2.2.2. Nano ZnO Film

When sputtering ZnO films on fabrics, the coated fabrics could get favorable anti-ultraviolet property, electrical conductivity and other properties [85,86]. Moreover, through improving its photocatalytic activity. The coated fabrics could obtain excellent antibacterial property [86]. Deng et al. [87] sputtered nano ZnO films on the surface of polyester non-woven fabric. With the increasement of sputtering time and power, the nano ZnO particles became larger, the film was uniform, and the coated fabric had favorable UV resistance. Boroujeni et al. [34] sputtered nano ZnO film on the surface of carbon fiber composite material. Its tensile strength was improved by 18% and it was obtained certain anti-ultraviolet property.

#### 2.2.3. ITO (Indium Tin Oxide) and AZO (Aluminum Doped Zinc Oxide) Films

ITO film is a kind of transparent conductive film [88]. AZO film is extremely highly electrically conductive and has a photoelectric property comparable to ITO. Deposited with ITO or AZO film, the coated fabrics can get favorable function of electrochromism, UV resistance, infrared resistance, electrical conductivity, hydrophilicity or hydrophobicity, etc. [89].

Sputtering ITO film exclusively can only provide a certain electrical conductivity and UV resistance properties, Beica et al. [90] sputtering deposited ITO film on glass fiber woven fabric, which obtained the properties mentioned above. 

Under certain conditions, AZO film can provide good waterproof property. For example, Jiang et al. [91] sputtered AZO film on polyester fabrics. When the thickness of the film was 450 nm, its contact angle (CA) was 146 degrees. Meanwhile, UV transmittance and infrared emissivity were greatly decreased.

More applications of ITO films are to make the substrates have electrochromic and electrically conductive property. For example, ITO, Pt, and WO_3_ films were sputtered on wool fabrics, respectively. The coated fabrics not only had electrically conductive and electrochromic properties, but also obtain excellent UV resistance property [92]. Or, the ITO film was firstly electrochemical deposited on the surface of polyester fabrics, and then the WO_3_ film was sputtering deposited on the surface of ITO film. The sputtering coated fabrics had electrochromic and electrical conductivity property [93].

#### 2.2.4. Other Nano Metal Oxides Films

Nano CuO films were sputtering deposited on polyester non-woven fabrics. The coated fabrics had strong antibiotic effect on *Escherichia coli* and *Staphylococcus aureus* [94].

Eren et al. [95] deposited nano V_2_O_5_ film on the surface of polyester fabric to prepare electrochromic fabric. With the increase of sputtering time, the films became continuous and compact. And the coated fabrics had favorable electrochromic properties.

### 2.3. Polymer Nano Film

Sputtered nano polymer films enable fabrics to obtain multiple functions. The most common sputtered polymer is polytetrafluoroethylene (PTFE) which can endow fabrics with anti-ultraviolet, waterproof and other performance [96,97].

PTFE has perfect hydrophobic performance. Huang et al. [98] deposited PTFE films on the surface of silk which make the coated fabrics become hydrophobic. The contact angle was increased from 68 degrees to 138 degrees. With the increase of sputtering pressure, the phenomenon of contact angle hysteresis became inconspicuous. In the same way, Wi et al. [99] deposited PTFE film on cotton fabric and the contact angle reached 134.2 degrees.

In addition, PET fabric sputtered with PTFE can also be UV resistant. The influence of substrate temperature, sputtering power, and sputtering pressure on UV resistance increased successively [100].

### 2.4. Multi-layer Coated Nano Film

Compared with single-layer film, multi-layer films can obtain multiple properties, such as UV resistance, antistatic, electromagnetic shielding property, antibacterial property and other properties. Miao et al. [101] sputtered AZO/Ag/AZO three-layer film on polyester fabric. When the Ag layer reached 10nm, a continuous film was formed, and its visible light transmittance was 80.5%. When the AZO (30 nm)/Ag (13 nm)/AZO (30 nm) structure was formed, the infrared reflectance was as high as 96%. In this research, properties of AZO/Cu/AZO films were compared with that of AZO/Ag/AZO films on polyester fabrics. At sputtering pressure of 100 Pa. The AZO/Cu/AZO three-layer film coated fabrics’ hydrophilicity was slightly improved (contact angle is reduced from 93.5 degrees to 88.5 degrees), UV resistance was slightly improved (UPF value increased from 40.64 to 48.437), and the infrared reflectance was reduced greatly, from 96 to 60% [102].

Multilayer composite films can also obtain structural color due to interference and diffraction of the films [103]. As the thickness of TiO_2_ increased, the sputtered fabric colors were different, such as purple (42 nm), light blue (66 nm), blue (82 nm), pink (98 nm) and deep red (115 nm) in Ag/TiO_2_ composite film on the surface of polyester fabric [17]. However, the researches on structural color through magnetron sputtering technology were less published at present. It mainly involved the generation of colors. Deeper researches on the extensiveness of structural color chromatography and the stability and reproducibility of structural colors had not been reported. More often, multi-layer films can achieve better effect than single layer films. Koprowska et al. [104] sputtered Cu/Sn and Cu/Zn/Ni films on the surface of polypropylene (PP) nonwoven fabrics. Ziaja et al. [105] sputtered Zn/Bi film and ZnO/Ti film on the surface of same fabrics. Both of them obtained favorable electromagnetic shielding property and good adhesion between the films and PP [106]. Rtimi et al. [107] also found that in Cu film, TiO_2_ film and TiO_2_/Cu composite film, antibacterial effect of TiO_2_/Cu composite film was much better than that of the others. It was suggested that TiO_2_ had a synergistic effect on Cu [108]. Similarly, the antibacterial efficiency of the Cu/CuO composite film sputtered on polyester fabric against *E. coli* was more than three times higher than that of the sputtered Cu film [109].

### 2.5. Effect of Plasma Pre-Treatment on Sputtering Coat

There is better adhesion between magnetron sputter coatings and fabrics compared to other methods. However, the adhesion between coatings and fabrics was discrepant due to sputtering processes and substrates different. For example, high-power impulse magnetron sputtering proceeded with a higher density of electrons/metalion pairs and at higher energies compared to direct current magnetron sputtering (DCMS) and direct current pulsed magnetron sputtering (DCMSP), thereby improved the adhesion between coatings and substrates [37]. For this kind of fabric substrate, the different surface chemical property, surface morphology and porosity size of the fiber materials can also cause differences in adhesion between coatings and substrates, besides the fabric structure differences such as knitted fabrics, woven fabrics and nonwoven fabrics, etc. When the adhesion was not good enough, it was possible to significantly improve the adhesion by appropriately improved the insufficient activation of fibers surface, internal stress, and differences in the thermal expansion coefficient [31]. Plasma pre-treatment of the fabric can increase adhesion between films and fabrics. The improving effect on adhesion was universal.

Before depositing a brass (Cu: Zn = 65:35, wt.%, Cu/Zn elemental composition ratio of 1.86) film on polyester fabric by high-power impulse magnetron sputtering (HIPIMS), Chen et al. [110] used oxygen plasma to pretreat the fabric for 1 min. The adhesion between brass film and fabric was increased obviously. When it was sputtered for 1 min, the coated fabric provided durable antibacterial properties against *Staphylococcus aureus* and *Escherichia coli*. Dry and wet rubbing fastness of the coated fabric can reach grade 5 and grade 4–5, respectively. Overall, the increased coating adhesion improves the color fastness of the pretreated fabric in rubbing, such that the coating reaches grade 5 during dry rubbing. The pre-treatment with oxygen plasma increases the film adhesion because it causes activation and the required PET surface functionalization. Such chemical importance brought about by oxygen plasma pre-treatment is crucial as well as physically cleaning the surface.

**Figure 5:**
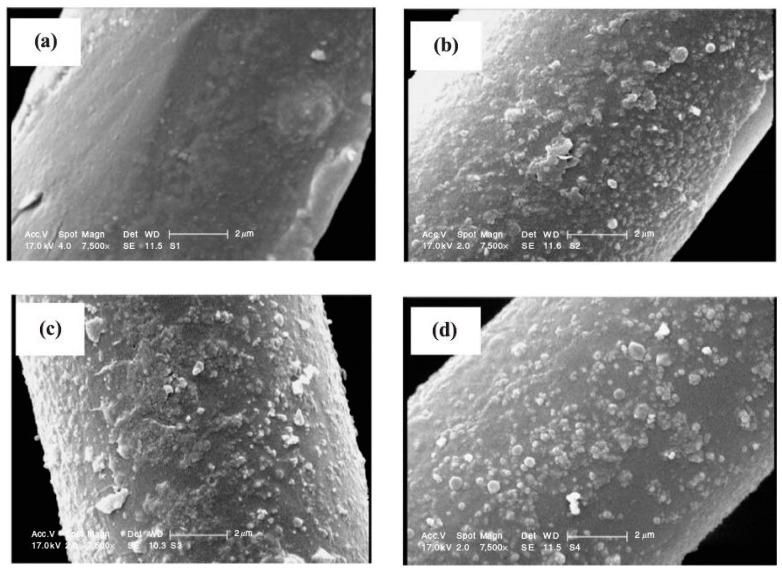
SEM images from surfaces that were plasma-treated with subsequent deposition. (**a**) Untreated; (**b**) 3 min plasma treated; (**c**) 5 min plasma treated; (**d**) 10 min plasma treated. Scale 2 μm (Reprinted from Reference [111] with permission).

Saffari et al. [111] used low temperature plasma to pre-treat polylactic acid (PLA) fabric before depositing TiO_2_ film on it. With the increase of plasma treating time and sputtering time, TiO_2_ particles on the PLA fibers’ surface became more compact. Figure 5 shows the scanning electron microscope image of the samples. When plasma pre-treatment was 10 min, the antibacterial and photo-catalytic properties of the coated fabric was best and can endure many times washing. The initially grown TiO_2_ film and the chemical modification that is caused by pretreatment with oxygen plasma clearly synergistically improved film adhesion to resist washing.

In other studies, for example, Wei et al. [112] deposited transparent ITO films on polyester nonwoven fabrics. Depla et al. [22] deposited Al_2_O_3_ films on the surface of polyester woven and non-woven ones, plasma pre-treatment all can significantly improve the adhesion of the coatings and their continuity and compactness.

## 3. Conclusions and Outlooks

Magnetron sputtering technology is a new-style method for the surface modification of textiles. The use of magnetron sputtering technology can impart textiles anti-static, antibacterial, anti-ultraviolet, electromagnetic shielding, electrical conductivity and other single or composite properties. The effect of interference and diffraction of nano-films even can also be used to obtain structural color effect. However, there are still many problems that require further research.

First, the functions of coated fabrics need to be further improved. For example: (1) How electromagnetic shielding fabrics achieve high effectiveness (≥60 dB); (2) How to achieve microwave electromagnetic shielding at a higher frequency range of 10–18 GHz; (3) How to achieve the selective shielding within the specified frequency range; (4) How antibacterial fabrics achieve low drug resistance, broad spectrum antibacterial properties and no toxicity.

Second, the papers on structural color coated fabrics have only been published in the past two years. The research is still not perfect and will probably be the hotspot and trend of future research. (1) How to achieve the extensiveness of structural colors’ chromatography; (2) How to make the structural color obtain favorable reproducibility and stability; (3) Characterization methods of structural color textiles and their various fastness. The structural color of coated fabrics is different from traditional dyeing. The fastness evaluation method applied to traditional dyeing cannot be applied to structural color. However, there is no applicable method for characterizing structural color fastness at present. So, further exploration is required.

Third, research on the safety of coated fabrics on the human body needs to be further strengthened. Since various metals and metallic compounds are required for the sputtering process, there are few studies on the effect of these nano-sized metals and metallic compounds on human health. There is an urgent need to strengthen research.

## Figures and Tables

**Table 1:** Common target materials and functions and characterizing methods of magnetron sputtering coated fabrics.

Serial Number	Target Materials	Functions	Characterizing Methods
1	Cu, Ag, Ti, Al, Ni, TiO_2_, ZnO, WO_3_, V_2_O_5_, Al_2_O_3_, ITO, AZO, etc.	Electromagnetic shielding, anti-static, conductive properties	Electromagnetic shielding efficiency, charge surface density, electrical conductivity, resistivity, square resistance
2	Cu, Ag, Ti, Al, Ni, Pt, TiO_2_, ZnO, MgO, ITO, AZO, PTFE, etc.	Anti-UV, anti-infrared properties	UPF (ultraviolet protection factor) value, infrared reflectivity
3	Cu, Ag, Zn, Ti, TiO_2_, Pt, ZnO, MgO, CuO, brass, etc.	Antibacterial property	Bacteriostatic rate
4	Ni, Al, TiO_2_, PTFE, TiN, SiO_2_, etc.	Hydrophobic and hydrophilic properties	Static contact angle, surface free energy
5	TiO_2_, ZnO, MgO, SnO_2_, Al_2_O_3_, Fe_2_O_3_, etc.	Adsorbed gas or gas sensitivity properties	Resistivity, electrical conductivity
6	Ti, Al, TiO_2_, WO_3_, SiO_2_, SnO_2_, TiN, etc.	Structural color effect	Reflectance, transmittance, refractive index
7	SiO_2_, Al_2_O_3_, LaB_6_, etc.	Heat resistant or warmness	Thermal stability, Thermal insulation coefficient, Thermal conductivity
8	Cu, Ag, Pt	Improved dyeing fastness	Light fastness, washing fastness
